# Self-reported sexually transmitted infections among sexually active men in Ghana

**DOI:** 10.1186/s12889-021-11030-1

**Published:** 2021-05-26

**Authors:** Abdul-Aziz Seidu, Ebenezer Agbaglo, Louis Kobina Dadzie, Justice Kanor Tetteh, Bright Opoku Ahinkorah

**Affiliations:** 1 Department of Population and Health, University of Cape Coast, Cape Coast, Ghana; 2 College of Public Health, Medical and Veterinary Sciences, James Cook University, Townsville, Queensland Australia; 3 Department of English, University of Cape Coast, Cape Coast, Ghana; 4 The Australian Centre for Public and Population Health Research (ACPPHR), Faculty of Health, School of Public Health, University of Technology Sydney, Ultimo, Australia

**Keywords:** STIs, Men, Ghana, Public health, Global Health

## Abstract

**Background:**

In sub-Saharan Africa, sexually transmitted infections (STIs) other than HIV are major public health problems. This study, therefore, sought to assess the prevalence and factors associated with self-reported STIsamong sexually active men in Ghana.

**Methods:**

Data from the 2014 Ghana demographic and health survey wereused to conduct the study. This research included a total of 3051 sexually active men aged 15–59 years. Self-reported STI was the outcome variable. The data were analyzed using both descriptive (frequencies and percentages) and inferential (binary logistic regression) analysis.

**Results:**

The prevalence of self-reported STIs in the past 12 months preceding the survey was 6.0% (CI:4.7–6.8). Compared to men aged 45-59 years, those aged 25–34 (aOR = 2.96, CI: 1.64–5.35), 15–24 (aOR = 2.19, CI: 1.13–4.26), and 35–44 (aOR = 2.29, CI: 1.23–4.24) were more likely to report an STI. Men who had 2 or more sexual partners apart from their spouse were more likely to report an STI compared to those with no other partner apart from spouse (aOR = 4.24, CI: 2.52–7.14). However, those who had their first sex when they were 20 years and above (AOR = 0.66, CI: 0.47–0.93) and men who read newspaper/magazine had lower odds (aOR = 0.53, CI: 0.37–0.77) of reporting STIs compared to those who had sex below 20 and those who did not read newspaper/magazine respectively.

**Conclusion:**

The study has revealed a relatively low prevalence of self-reported STI among sexually active men in Ghana. Sexually active men aged 25–34 years, those whose age at first sex is below 20 years and those with two or more sexual partners apart from their spouse had higher odds of reporting STIs. However, reading a newspaper was found to be positive in reducing the odds of reporting STIs. To reduce STIs among sexually active men in Ghana, it is important for health systems and stakeholders to consider these factors and put in place measures to mitigate those that put men at risk of STIs and encourage the adoption of the protective factors. Mass media can be used as a useful avenue for encouraging men to report STIs in order to avoid transmitting them to their partners.

## Background

Sexually transmitted infections (STIs), also known as sexually transmitted diseases (STDs), comprise a number of diseases that are transmitted from one person to another through sexual intercourse [1–4]. They are part of the most common acute health conditions [1, 4]. While there are about 30 STIs, the commonest ones include vaginosis, herpes, chlamydia, trichomoniasis, gonorrhoea, hepatitis B virus, and syphilis [2]. Among these, chlamydia, gonorrhoea, syphilis, and trichomoniasis are curable [1, 3]. STIs have both health and psychological effects, such as genital symptoms, infertility, and enhanced HIV transmission [4]. Globally, the prevalence of STIs remain high. The prevalence of new infections of the four curable STIs rose from 359 million in 2012 [1] to 376 million in 2016 [3]. STIs and their complications are among the top five disease groups for which adults seek medical attention in low- and middle-income countries [3]. Other than HIV, STIs are a big public health problem in sub-Saharan Africa [2].

STIs pose a serious threat to the international community. Due to this, the WHO adopted a strategy in 2016 aimed at ending STIs [3]. Among the targets of the strategy is to increase information on STIs. This requires research to reveal the global burden of the disease [4]. Previous studies on STIs in sub-Saharan African countries have focused on the region as a whole [4] and on specific countries such as Ghana [5–7] and Ethiopia [2]. Such studies have identified religion, mass media exposure, and having multiple partners as predictors of STIs. In Ghana, such research has mainly focused on women [7–10]. In this present study, we sought to determine the prevalence and assess the factors associated with self-reported STIs among sexually active men in Ghana. Results emanating from this study are vital since theywill be a major contribution to the existing literature on STIs in Ghana. Additionally, such information will be relevant to interventions such as education and counselling targeted at fighting against STIs.

## Methods

### Study design and data source

We sourced data from the 6th version of the Ghana Demographic and Health Survey (GDHS). The GDHS is a nationally representative survey that is mostly conducted in 5 year intervals in over 85 low and middle income countries globally. In Ghana, the first round was conducted in 1988, the second one in 1993, third in 1998, fourth in 2003, fifth in 2008 and the most recent one is the 2014 version. The 2014 version covered all the then ten regions (now sixteen) in Ghana. The 2014 GDHS was implemented by the Ghana Statistical Service and the Ghana Health Service with Inner City Fund International giving technical support through MEASURE DHS [11, 12]. The survey gathers information on a variety of topics, including maternal and child health, to track key maternal and child health indicators in low- and middle-income countries. The survey also collects data from men aged 15–59 or 64 years in some countries. The DHS’s sampling technique is a multistage procedure. For example, in the 2014 GDHS the first stage is marked by the collection of clusters made up of enumeration areas drawn up for the 2010 Census of Population and Housing [[11] p317–319]. The clusters were selected from the previous 10 administrative regions in Ghana containing both urban and rural areas. With the second stage households are selected from each cluster. Details of the methodology has been documented elsewhere [11] p317–19]. In the 2014 DHS, 4388 men aged 15 to 59 were interviewed, with 3,498 admitting to having ever had sex. However, for this analysis, 3,051 sexually active men were considered, all of whom had full details on all of the variables of interest.

### Variables

#### Dependent variable

The dependent variable was self-reported STIs. It was derived from men’s self-reporting of STI in the 12 months prior to the study. Men were asked if they had contracted a disease through sexual intercourse in the previous 12 months, and they were given two options: Yes or No [2, 12].

#### Independent variables

Independent variables included in the analysis were age (years) (15–24,25-34,35-44,44+), residence (rural, urban), ecological zone (northern, middle, coastal), educational level (no education, primary, secondary/higher), wealth status (poor, middle, rich), marital status (married, not married), religion (Christianity, Islam, Other), ethnicity (Akan, Ga/Dangme/Ewe, Mole Dagbani, Other), employment status (working, not working), age at first sex (years) (<=19, 20 and above), number of sexual partners in the last 12 months apart from regular partner (0,1, 2+), HIV testing (Yes, No), exposure to mass media (newspaper/magazine, radio, television) (Yes, No) and health insurance coverage (Yes, No) (see Table 1). We chose these factors due to their theoretical and empirical association with STIs in previous studies [1, 2, 12].

**Table 1:** Socio-demographic characteristics of the study participants and prevalence of self-reported sexually transmitted infections across independent variables

Variables	Weighted sample (***n*** = 3051)		Had any STI in the last 12 months	95%CI	
	Frequency	Percentage	Yes (%)	Lower bound	Upper bound
**Age (years) (**χ^2^ **= 48.9,** ***p*** **< 0.001)**					
15–24	501	16.41	9.8	7.63	14.24
25–34	961	31.49	7.83	5.67	9.92
35–44	839	27.48	4.4	3.07	6.28
45+	751	24.62	1.79	0.90	2.82
**Residence (**χ^2^ **= 0.75,** ***p*** **= 0.385)**					
Urban	1594	52.23	5.29	3.87	6.57
Rural	1457	47.77	6.01	4.98	8.17
**Ecological Zone (**χ^2^ **= 13.8,** ***p*** **= 0.001)**					
Northern zone	379	12.43	3.27	1.78	4.37
Middle zone	1449	47.5	7.12	5.28	8.17
Coastal zone	1223	40.07	5.61	3.95	7.70
**Education (**χ^2^ **= 13.5,** ***p*** **= 0.001)**					
No education	375	12.3	2.47	1.15	4.13
Primary	390	12.77	5.2	3.65	8.66
Second/higher	2286	74.93	6.58	5.12	7.66
**Wealth index (**χ^2^ **= 5.1,** ***p*** **= 0.079)**					
Poor	973	31.89	4.82	3.43	6.49
Middle	624	20.46	7.38	5.25	9.77
Rich	1454	47.65	5.72	4.28	7.53
**Marital status (**χ^2^ **= 56.8,** ***p*** **< 0.001)**					
Not married	1291	42.31	9.59	7.83	11.89
Married	1760	57.69	3.13	2.04	3.77
**Religion (**χ^2^ **= 1.7,** ***p*** **= 0.429)**					
Christianity	2206	72.3	5.96	4.76	7.22
Islam	521	17.07	4.6	2.72	6.46
Other	324	10.62	5.88	4.36	10.62
**Ethnicity (**χ^2^ **= 9.8,** ***p*** **= 0.020)**					
Akan	1497	49.07	6.71	4.99	8.35
Ga/Dangme/ewe	720	23.59	6.72	4.56	8.73
Mole Dagbani	417	13.67	3.89	2.42	6.37
Other	417	13.67	4.22	2.22	5.63
**Occupation (**χ^2^ **= 0.2,** ***p*** **= 0.667)**					
Not working	117	3.83	4.8	2.39	13.22
Working	2934	96.17	5.71	4.72	6.84
**Age at first sex (years) (**χ^2^ **= 21.2,** ***p*** **< 0.001)**					
< =19	1705	55.9	7.45	6.15	9.31
20 and above	1346	44.1	3.57	2.34	4.63
**﻿ Number of sex partners excluding spouse in the last 12 months (**χ^2^ **= 123.9,** ***p*** **< 0.001)**					
0	1790	58.68	3.14	2.08	3.84
1	1008	33.03	7.11	5.37	9.25
2+	253	8.3	21.2	14.46	28.17
**Ever been tested for HIV (**χ^2^ **= 2.9,** ***p*** **= 0.108)**					
No	2250	73.76	6.06	5.19	7.71
Yes	801	26.24	4.52	2.66	5.68
**Exposure to Mass media**					
**Newspaper/magazine (**χ^2^ **= 5.04,** ***p*** **= 0.025)**					
No	1993	65.33	6.29	5.60	8.15
Yes	1058	34.67	4.24	2.50	5.35
**Radio (**χ^2^ **= 0.82,** ***p*** **= 0.364)**					
No	127	4.15	4.0	1.03	5.56
Yes	2924	95.85	5.76	4.85	7.01
**Television (**χ^2^ **= 8.2,** ***p*** **= 0.004)**					
No	469	15.38	3.28	2.10	5.86
Yes	2582	84.62	6.27	5.02	7.36
**Covered by NHIS (**χ^2^ **= 2.5,** ***p*** **= 0.114)**					
No	1618	53.04	6.34	5.12	8.24
Yes	1433	46.96	5.01	3.60	6.20
STI Prevalence			6.00	4.70	6.80

**Source: 2014 GDHS**

*CI* confidence interval

### Statistical analyses

Stata 14.0 was used to conduct all of the analyses (Stata Corporation, College Station, TX, USA). To analyze our data, we used both descriptive and inferential statistics. We used frequencies and percentages to classify the sample using descriptive statistics. To pick possible variables for the multivariable logistic regression analysis, a Chi-square test was used. The multivariable binary logistic regression model was used to model  variables that had a chi-square test *p*-value of less than 0.05. Before fitting the final model, the variance inflation factor (VIF) was used to determine multi-collinearity among the independent variables (Mean VIF = 1.45, Minimum = 1.09, Maximum VIF = 2.03), and no evidence of collinearity was found. Adjusted odds ratios (aORs) with 95% confidence intervals (CIs) and *p*-values were used to present the regression results. Statistical significance was declared at *p* < 0.05 in all of the analyses. Using the sample weight (v005/1,000,000), the data were weighted to account for the complex sampling structure and non-response. The data was also adjusted using the ‘svy’ command to account for the complex survey nature. 

## Results

### Socio-demographic characteristics of the study participants and prevalence of sexually transmitted infections across the independent variables

In Table 1, we present the socio-demographic characteristics of the respondents and STI prevalence. Slightly more than 30 % of the respondents were aged 25–34. More than half (52.2%) were in the urban areas, 47.5% were in the middle ecological zone, 74.9% had secondary/tertiary level of education, 47.7% were in the rich wealth index, 57.7% were married and 72.3% were Christians. Almost 60% of the participants (58%) were married. Almost half of the study participants (49%) were of the Akan ethnic group. Almost all the participants (96%) reported being employed whereas 55.9% had their first sex during adolescence. Out of a total of 3051 study participants, 174 representing 6.0% (CI = 4.7–6.8), reported to have contracted STI within 12 months preceding the survey. The chi-square analysis also showed that ecological zone, education, marital status, ethnicity, age at first sex, number of sexual partners in the past 12 month and exposure to television had statistical significance with self-reported STIs.

### Logistic regression analysis results on the factors associated with self-reported STI among sexually active men in Ghana

Table 2 presents adjusted odds ratio at 95% confidence level on the factors associated with STI among sexually active men in Ghana. Compared to men aged 45–59, those aged 25–34 (aOR = 2.96, CI:1.64–5.35), 15–24 (AOR = 2.19, CI: 1.13–4.26) and 35–44 (AOR = 2.29, CI: 1.23–4.24) were more likely to report STI. Respondents who had their first sex when they were 20 years and above had lower odds of reporting STIs compared to those who had their first sex at a younger age (aOR = 0.66, CI: 0.47–0.93). Respondents who had 2 or more sexual partners were more likely to report an STI (aOR = 4.24, CI: 2.52–7.14) as compared to their counterpart who had one or no sexual partner. Men who read newspaper had lower odds (aOR = 0.53, CI: 0.37–0.77) of reporting STI compared to their counterparts who reported that they were not reading newspaper.

**Table 2:** Factors associated with self-reported sexually transmitted infections among sexually active men in Ghana

Variables	aOR	95% CI		***P***-value
Age (years)		Lower bound	Upper bound	
15–24	2.195	1.13	4.26	0.020
25–34	2.96	1.64	5.35	*P* < 0.001
35–44	2.29	1.23	4.24	0.009
45–59	Ref	Ref	Ref	Ref
**Ecological Zone**				
Northern zone	Ref	Ref	Ref	Ref
Middle zone	1.59	0.86	2.93	0.142
Coastal zone	1.12	0.58	2.17	0.730
**Educational level**				
No education	0.72	0.39	1.35	0.304
Primary	0.75	0.46	1.18	0.209
Higher	Ref	Ref	Ref	Ref
**Marital status**				
Not married	Ref			
Married	0.77	0.47	1.28	0.311
**Ethnicity**				
Akan	1.02	0.59	1.76	0.951
Ga Adangme/Ewe	1.33	0.72	2.45	0.353
Mole Dagbani	1.04	0.58	1.86	0.890
Other	Ref	Ref	Ref	Ref
**Age at first sex (years)**				
< =19	Ref	Ref	Ref	Ref
20 and above	0.66	0.472	0.926	0.016
**﻿Number of sex partners excluding spouse in the last 12 months**				
0	Ref	Ref	Ref	Ref
1	1.51	0.94	2.412	0.087
2+	4.238	2.52	7.14	*P* < 0.001
**Exposure to mass media**				
**Newspaper/Magazine**				
No	Ref	Ref	Ref	Ref
Yes	0.53	0.37	0.77	*P* < 0.001
**Television**				
No	Ref	Ref	Ref	Ref
Yes	1.45	0.88	2.38	0.144
N	3051			
**R**^**2**^	**0.107**			

*Ref* Reference category *CI* Confidence Interval

Source: 2014 GDHS

## Discussion

In sub-Sahara Africa, the top five disease groups for which adults seek health treatment includes STIs and their related complications [2]. This study sought to assess the prevalence and factors associated with self-reported STIs among sexually active men in Ghana. The prevalence of self-reported STIs in this study was 6% which is higher than 3.5% prevalence reported in Ethiopia by Dagnew, Asresie and Fekadu [2] and SSA [12] but slightly lower than the prevalence of 7.4% in Kenya reported from a national population-based survey by Oluoch et al. [13].

It was also found that men in the ages of 15–44 were more likely to report STI, compared to those aged 45–59, with those aged 25–34 having the highest odds of self reporting STIs. Evidence suggests that young people in Ghana engage in risky sexual behaviours including sex without condoms and this might predispose them to the contraction of STIs [14, 15]. We further observed that age at first sex was significant in predicting the odds of STIs. Men who had their first sex after adolescence stage had lower risk of STI compared to those who had their first sex during the adolescence stage. This finding supports findings from previous studies in China [16], Tanzania [17], Lesotho [18], Malawi [19], and in Republic of Korea [20]. Evidence in other parts of sub-Saharan Africa and Ghana suggest that adolescence period is one that is associated with several risky sexual behaviours including multiple sexual partners, experimenting with sex, sex without condom and alcohol use during sexual intercourse [15, 21]. All these factors might predispose those who had their sex at that stage of their life to STIs.

We also found that among Ghana’s sexually active men, the number of sexual partners was linked to self-reported STI. Men who had two or more sexual partners other than their spouses were more likely to report a STI infection than men who had no sexual partners other than their usual partners. This finding supports findings of studies conducted in Ethiopia [2, 22], South Africa [23], Kenya [24] and sub-Saharan Africa [12]. Studies have shown that STI testing in many sub-Saharan African countries are low and people are more likely to spread infections without their knowledge [25]. We therefore infer from the finding that the more sexual partners people have, the increased likelihood that one of them might already be infected and may transfer the infection to the others.

Exposure to mass media also showed statistically significant relationship with self-reporting STI. Specifically, those who read newspaper/magazine had lower odds of reporting STIs compared to their counterparts who did not. Studies have reported that access to mass media education such as newspapers positively affect ones behaviour towards unhealthy sexual practices [26, 27]. This study’s results are consistent with those of a cross-sectional study in Ethiopia [2].

### Strength and limitation

The study’s key strength is its use of a nationally representative dataset with a relatively large sample size. In terms of methodology, the DHS survey follows best practices, and this, combined with the use of experienced and well-trained data collectors, resulted in a high response rate. As a result, the findings of this study can be extended to all Ghanaian men who are sexually active. Despite these strengths, the cross-sectional study design prevents causal inference from the findings. Self-reports were used to measure the outcome variable, which were not checked or validated by any medical professional. Since many of the most common STIs are asymptomatic (most notably chlamydia and trichomoniasis in men), self-reporting is often restricted because asymptomatic infections are not recorded. It is also possible that some of the factors we controlled for (e.g., education, wealth index) may have affected symptom recognition or STI in a way that cannot be controlled for without biological testing. In addition, there is also the likelihood of recall and social desirability biases [28]. Furthermore, the type of STI was not specified [12].

## Conclusion

The study has shown a relatively low prevalence of self-reported STIs among sexually active men in Ghana. This prevalence varied across the socio-demographic characteristics of the men. Age, age at first sex, number of sexual partners and exposure to newspaper are the factors associated with self-reported STIs among sexually active men in Ghana. Specifically, sexually active men in the ages of 25–34, those whose age at first sex is below 20 years and those with two or more sexual partners had higher odds of contracting STIs. Reading newspaper/magazine however was found to be positive in reducing the odds of contracting STIs. It is, therefore, recommended that to reduce STIs among sexually active men in Ghana, it is important for health systems and stakeholders to consider these factors and put in place measures to mitigate those that put men at risk of STIs and encourage the adoption of the protective factors. Mass media can be used as a useful avenue for encouraging men to report STIs in order to avoid transmitting them to their partners. 

## Data Availability

The dataset used and/or analysed during the current study is available from the corresponding author on reasonable request.

## Abbreviations

AOR: Adjusted odds ratio
CI: Confidence interval
GDHS: Ghana demographic and health survey
STI: Sexually transmitted infections
